# Identification of potentially functional modules and diagnostic genes related to amyotrophic lateral sclerosis based on the WGCNA and LASSO algorithms

**DOI:** 10.1038/s41598-022-24306-2

**Published:** 2022-11-22

**Authors:** Narges Daneshafrooz, Masumeh Bagherzadeh Cham, Mohammad Majidi, Bahman Panahi

**Affiliations:** 1 Department of Neuroscience, Faculty of Advanced Technologies in Medicine, Iran University of Medical Science, Tehran, Iran; 2 School of Rehabilitation Science, Iran University of Medical Science, Tehran, Iran; 3 Neuromusculoskeletal Research Center, Physical Medicine and Rehabilitation Department, Firoozgar Hospital, School of Medicine, Iran University of Medical Science, Tehran, Iran; 4 Department of Biotechnology, Faculty of Agriculture, Azarbaijan Shahid Madani University, Tabriz, Iran; 5 Department of Genomics, Branch for Northwest & West Region, Agricultural Biotechnology Research Institute of Iran (ABRII), Agricultural Research, Education and Extension Organization (AREEO), Tabriz, Iran

**Keywords:** Computational neuroscience, Neurology

## Abstract

Amyotrophic lateral sclerosis (ALS) is a genetically and phenotypically heterogeneous disease results in the loss of motor neurons. Mounting information points to involvement of other systems including cognitive impairment. However, neither the valid biomarker for diagnosis nor effective therapeutic intervention is available for ALS. The present study is aimed at identifying potentially genetic biomarker that improves the diagnosis and treatment of ALS patients based on the data of the Gene Expression Omnibus. We retrieved datasets and conducted a weighted gene co-expression network analysis (WGCNA) to identify ALS-related co-expression genes. Functional enrichment analysis was performed to determine the features and pathways of the main modules. We then constructed an ALS-related model using the least absolute shrinkage and selection operator (LASSO) regression analysis and verified the model by the receiver operating characteristic (ROC) curve. Besides we screened the non-preserved gene modules in FTD and ALS-mimic disorders to distinct ALS-related genes from disorders with overlapping genes and features. Altogether, 4198 common genes between datasets with the most variation were analyzed and 16 distinct modules were identified through WGCNA. Blue module had the most correlation with ALS and functionally enriched in pathways of neurodegeneration-multiple diseases’, ‘amyotrophic lateral sclerosis’, and ‘endocytosis’ KEGG terms. Further, some of other modules related to ALS were enriched in ‘autophagy’ and ‘amyotrophic lateral sclerosis’. The 30 top of hub genes were recruited to a LASSO regression model and 5 genes (BCLAF1, GNA13, ARL6IP5, ARGLU1, and YPEL5) were identified as potentially diagnostic ALS biomarkers with validating of the ROC curve and AUC value.

## Introduction

Amyotrophic lateral sclerosis (ALS) is an adult-onset neurodegenerative disorder. This motor neuron disease is characterized by rapidly progressive paralysis, atrophy, and respiratory failure leading to death typically within 1 to 5 years after symptoms onset. The incidence of ALS is approximately 2 to 3 per 100,000 people each year^1^. When symptoms are beginning, it is difficult to distinguish ALS from other disease especially for those with motor neuron signs. Consecutive years the El Escorial criteria, which essentially rely on motor signs, is using for ALS diagnosis and progression. However, it has estimated a false-positive of 8–10% and a false-negative of approximately 45% for applying this measure to evaluate ALS patients^2^.

ALS appears in 5–10% of cases with family history (fALS), while almost 90% of ALS patients are affected without family history (sALS). Genetic studies have revealed more than 30 genes contributing in ALS among which, C9orf72, SOD1, TARDBP, and FUS are more frequently involved^3^. Increasing evidence suggests that several ALS related genes represent a considerable phenotypic pleiotropy. Previous studies indicated mutations in some identical genes including C9orf72, TARDBP, FUS, CHCHD10, UBQLN2, SQSTM1, VCP and TBK1 have associated with the both ALS and FTD. Furthermore, C9ORF72 variants have been detected in Parkinson’s disease or Alzheimer’s dementia patients. TBK1 and KIF5A linked to several other neurodegenerative diseases beyond ALS, especially to cerebellar ataxia syndrome and hereditary spastic paraplegia, respectively^4,5^.

Despite the known genes related to ALS, the precise cause and underlying mechanism have not completely elucidated. Vast majorities of efforts are made in multiple areas to determine an acceptable biomarker as if meanwhile explain the pathogenic mechanism of ALS disease. Transcriptomics, a method to reveal differences in gene activity through mRNA expression, has been used in assessing the differential expression of mRNA in several disorders including neurodegenerative diseases. During recent years, transcriptome analysis provides means to explore and determine underlying molecular and cellular processes using high throughput technologies especially gene expression microarray and RNA-seq transcriptome profiling^6–11^. Nowadays, there is no standard approach for transcriptomic analyses, however network analysis is a novel strategy to analyze intricate datasets and to research the complex and heterogeneous diseases such ALS. Weighted gene co-expression network correlation-based analysis (WGCNA) is a precise approach of network analysis that assigns interconnected genes to the groups called ‘modules’, determines the most representative genes of each module as ‘eigengene’, and links the modules of co-expressed genes to a clinical phenotype^12–14^. In addition, WGCNA provides the investigation of the degree of module preservation in other datasets. In this study, we used WGCNA to Meta-analyze the previously published circulating transcriptome datasets of ALS and control subjects.

To date, circulating mRNA profiling of ALS patients through microarray and RNA-seq have applied to identify disease biomarkers, although the limited sample size of these studies decreases the reliability of their results. More recently, a large cohort dataset containing 1117 participants (GSE112681) has been generated by et al.^15^ and re-analyzed by others^16^. They used different strategies to identify different expressed genes that produced poor overlap results, while the identified DEGs did not present significant changes in expression (FDR < 0.1, fold change > 1.1 and < 9.9 for up regulated and down regulated DEGs, respectively).

In recent years, WGCNA has been used to identify circulating mRNA biomarkers in several conditions, including neurodegenerative diseases. In present study we conducted an integrated analysis of the blood transcriptome among ALS patients to identify circulating hub genes using weighted gene co-expression network analysis (WGCNA), a free R package, frequently used to explore the complex relationships between genes and phenotypes via simultaneously constructing gene networks and detecting gene modules and identifying the hub genes as central players within modules^17^. The most important advantage of WGCNA is transforming gene expression data into co-expression modules, providing insights into signaling networks that may be responsible for phenotypic traits of interest^18^. Here, co-expression network on gene expression datasets using WGCNA was employed to improve the level of significance in understanding biological mechanisms, including those related to diseases. Subsequently, we established a diagnostic-related gene signature set associated with ALS using logistic regression and least absolute shrinkage and selection operator (LASSO) analysis. The obtained model was verified using a receiver operating characteristic (ROC) curve.

One of the main challenges in determining biomarkers for ALS is finding a marker that can differentiate between ALS and some disorders with close symptoms. For example, FTD has been represented as one of two extremes of the ALS-FTD spectrum^19^. The discovery of shared genetic disorders and the subsequent common pathological pathways links ALS and FTD in a number of ways. Ongoing evidence from epidemiological studies imply that 30–50% of patients with ALS diagnosis have developed cognitive/behavioral impairments during the course of disease^20^. Further, there are 10 to 30% of patients with FTD who have demonstrated a varying range of a few motor neuron dysfunction symptoms up to the diagnosis of ALS^21^. On the other hand, ALS patients have symptoms of motor dysfunctions at initial presentation that may confuse clinicians in distinguishing ALS from other motor diseases with similar symptoms.

Therefore, a biomarker with capability of differentiating ALS from diseases with similar early symptoms would certainly be more efficient. Here, we evaluated the different patterns of gene co-expression between ALS and ALS-mimic diseases using a dataset containing gene expression profiling of patients with ALS mimicking symptoms. Besides, we sought to find the genes capable of discriminating ALS genotypes from FTD by analyzing a dataset of gene expression GSE140830^22^.

## Methods

### Dataset retrieval

The datasets GSE112681; including datasets GSE112676 and GSE112680, and GSE140830 were downloaded from the GEO database (http://www.ncbi.nlm.nih.gov/geo/). These datasets generated from whole blood gene expression profiling via platforms Illumina HumanHT-12 V3.0 (the first) and HumanHT-12 V4.0 expression BeadChip arrays (two latter), respectively. Since the raw data *idat* files were not deposited at GEO, we got expression data including *intensity* and *detection P-value* as supplementary files. Expression information was extracted using the *GEOquery* R package (Version, 2.58.0). Sample information was extracted from the *series matrix* file. Subsequently, for each dataset, the respective ‘preQC-nonnormalized.txt’ files were processed via *limma* R package (version 2.9.8).

Correcting platform effects using algorithms aimed to eliminate batch effects directly modifies data, and inevitably, statistical tests after batch effect removal will be conducted on modified data. Therefore, we decided to select one of the platforms as a training set and the other as a validation set instead of platform effect removal.

### Probe filtering and data processing

In both platforms, the probes that met the following criteria were excluded: (1) probes whose detection was statistically significant (p < 0.05) in at least three samples. (2) Control probes and those with no symbol, (3) probes that are not referring to protein-coding genes; those without ‘NP’ or ‘NM’ prefix in RefSeq identifier in annotation files, (4) Probes with more than 10% ‘NA’ value of samples. A microarray annotation file was used to assign probes with corresponding gene symbols. Probes with more than one gene symbol were eliminated and the average value was calculated out for those genes corresponding to more than one probes. Finally, the overlapped gene symbols between the datasets with CV > 20% remained as expression data matrices.

### Weighted gene correlation network analysis

The network co-expression analysis was performed using the *WGCNA* R package (version 1.71). The *flashClust* package (version 1.01-2) was used to perform the cluster analysis of samples with the appropriate threshold value, followed by plotting the sample clusters against clinical traits. Soft-thresholding powers tested ranging from 1 to 20 and the appropriate power value was determined. The co-expression similarity matrix calculated just as Pearson test for all pairs of genes and then transformed into an adjacency matrix by soft-thresholding power (β) as the smallest value to retain scale free topology. The adjacency matrix was then converted to a topological overlap matrix (TOM) for prospecting the modular structures of the co-expression network. The topological overlap dissimilarity, then was calculated using TOM, followed by constructing the gene clusters by hierarchical clustering in order to identify separated modules of interconnected genes. Subnetworks identified as co-expression modules are highly interconnected geneclusters that would be the true representatives of interactive genes contributing to biological pathways. Then, the module eigengene (ME), as the first principal component of each module was calculated. The ME is an expression representative of all genes in one module. Then, the module membership as a correlation between ME and gene expression pattern of a module was calculated. Finally, the gene significance (GS) of each gene in the module, which represented the correlation between gene and the clinical trait, was further calculated.

### Network preservation analysis

To evaluate the module preservation, we applied a Z-summary permutation testing using the *modulePreservation()* function in the *WGCNA* package. In addition to the module preservation test between the training and validation datasets, we conducted the testing to find the modules not preserved in diseases mimicking ALS and FTD patients based on data presented in datasets GSE112680 and GSE140830.

As mentioned above, the heterogeneity of ALS can be considered in both genotypic and phenotypic overlapping with other diseases such as several movement disorders and frontotemporal dementia. Obviously, there are similarities in gene expression profiles due to the common underlying pathological pathways involved in neurological and neurodegenerative diseases. Finding the co-expressed genes not correlated to ALS, can distinguish it from disorders that have mechanisms in common with ALS.

### Pathway enrichment analysis

To characterize the function of the significant gene modules, we performed KEGG pathway enrichment analysis^23^ using *clusterProfiler* and *DOSE* packages^24,25^. The threshold level of q-value < 0.05 were employed to indicate a significance for KEGG pathways.

### Visualization of hub genes using protein–protein interaction network

The hub genes of main modules with MM > 0.8 and GC > 0.5 were imported into the STRING database (https://www.string-db.org/) and protein‒protein interaction (PPI) networks were built. The results then were exported to Cytoscape Version 3.9.1 to visualize the PPI network. The CytoHubba app in Cytoscape was used to calculate the degree of each node and screen out the top hub genes.

### Screening and validation of hub genes

The gene signature for the prediction of ALS was performed through least absolute shrinkage and selection operator (LASSO) logistic regression analysis. In order to validate the hub genes acquired from the WGCNA method, the LASSO was conducted using the *cv.glmnet()* function (with tenfold cross-validation and alpha = 0.5) in R. The Lasso model shrinks the less important feature’s coefficient to zero with the aim of retaining the good features. By L1 regularization, the LASSO regression adds a penalty equal to the absolute value of the magnitude of coefficients. It can minimize the coefficients and yield a spares model. The strength of the penalty term in Lasso regression is controlled by a tuning parameter λ. When λ = 0, no coefficients (features) are eliminated. By increasing the value of λ, the more coefficients are set to zero and eliminated. In the present study, the hub genes of blue and black modules with gene significance > 0.5 and module membership > 0.8 were validated using this approach.

Subsequent to identifying genes that could potentially be diagnostic markers of ALS, receivers operating characteristic (ROC) curve analyses were conducted with the *pROC* R package. The area under the ROC curve (AUC) values were utilized to assess the predictive utility of identified key predictive genes. The AUC is a criterion for estimating the probability that a classifier (predictor), i.e. gene-set can predict better than randomly selected classifiers^26^.

## Results

### Demographic information of participants

The cohort included three large datasets, GSE112676, GSE112680, and GSE140830 with two platforms. GSE112676 contains 233 ALS diseases and 508 control subjects, while GSE112680 contains 164 ALS diseases, 137 control subjects, and 75 ALS-mimic diseases. GSE140840 consists of 172 FTD diseases (including 82 bvFTD, 46 nfvPPA, and 44 svPPA) and 281 control subjects. The phenotypic data (demographic data and clinical assessments) extracted from *series matrix* files, were contained diagnostic and sex information of all samples, in addition of site of onset, age of onset and duration of disease, as well as genetic state of C9orf72 for ALS patients. The sample information has been summarized in Supplementary Table S1.

### Gene expression data processing

Gene expression and annotation data were retrieved through the *GEOquery* R package. Gene expression data included raw expression intensities and detection P-values for all three datasets. Using *data.table* and *limma* packages, the raw expression and annotation files converted to matrices are appropriate for further analysis. The *neqc()* function was then used to apply a normal-exponential convolution model for background correction followed by quantile normalization and log2 transformation to the intensities. The control probes, those with no symbol, and those that failed to detect expression were filtered out. Finally, only probes remained that referring protein-coding genes; those with ‘NP’ or ‘NM’ prefix in RefSeq identifier in annotation files. Following the last filtering step, GSE112676, GSE112680, and GSE140840 datasets included 35,000, 33,765, and 31,980 probes, respectively. Values for replicate probes were then replaced with their average using the *avereps()* function.

Identifying the outliers is a necessary step before sample clustering. So, the expression values upper and lower than 1.5 times the interquartile range (IQR) were equalized to ‘NA’ in final matrices of expression data. The probes of > 10% ‘NA’ were excluded as outliers. After averaging and outlier removal, the datasets GSE112676, GSE112680, and GSE140830 contained 22,691, 24,818, and 23,574 genes, respectively.

When network analyzing, the genes with the most variation in expression provides more information, so we selected a set of genes with coefficient of variation (CV) ≥ 0.2. Then, only the genes overlapped between three datasets remained for following network analysis. Consequently, either datasets contained 4198 common genes.

### Removing unwanted variance

Batch effects, technical variation and other sources of unwanted variation are ever-present in big data, especially so in high-throughput gene expression profiling. Accordingly, we found an obviously inter-platform batch effect in three datasets using the principle component analysis (PCA) (Supplementary Fig. 1). Various algorithms that try to neutralize batch effects directly modify the data, therefore, preparing the data in a separate step of the analysis may lead to artifacts that could affect the reliability of the analysis. So, instead of correcting batch effects for meta-analysis, we decided to analyze GSE112676 as a training set and the GSE112680 as a validation set to compare the gene co-expression between ALS and control subjects. As well, comparing the gene co-expression between ALS and ALS mimic patients was possible by preservation analysis of these datasets. Further, by preservation analysis of dataset GSE140830, we managed to compare the gene co-expression between ALS and FTD patients.

We also evaluated the intra-platform batch effects in the datasets separately and found out an intra-platform batch effect in the GSE112676 dataset (Supplementary Fig. 2a). Furthermore, there was a direct correlation between the GSM indices and PC1 (cor = 0.69, P = 2.2e−16) (Supplementary Fig. 2b).

There are multiple ways to eliminate such variations including *empiricalBayesLM()* functions in the *WGCNA* package*.* So we applied empirical Bayes moderated linear model adjustment in function *empiricalBayesLM()* function. By applying the PCA on adjusted data, the batch effect, in addition to correlation between PC1 and GSM indices were removed (Supplementary Fig. 2d, c).

### Weighted-gene co-expression network analysis

After removing unwanted variances for the known source (GSM indices), the WGCNA procedure was used to construct the co-expression network and to identify the co-expressed modules in dataset GSE112676 (233 ALS and 508 control). We selected the GSE112676 as a training dataset because of the larger sample size.

**Figure 1:**
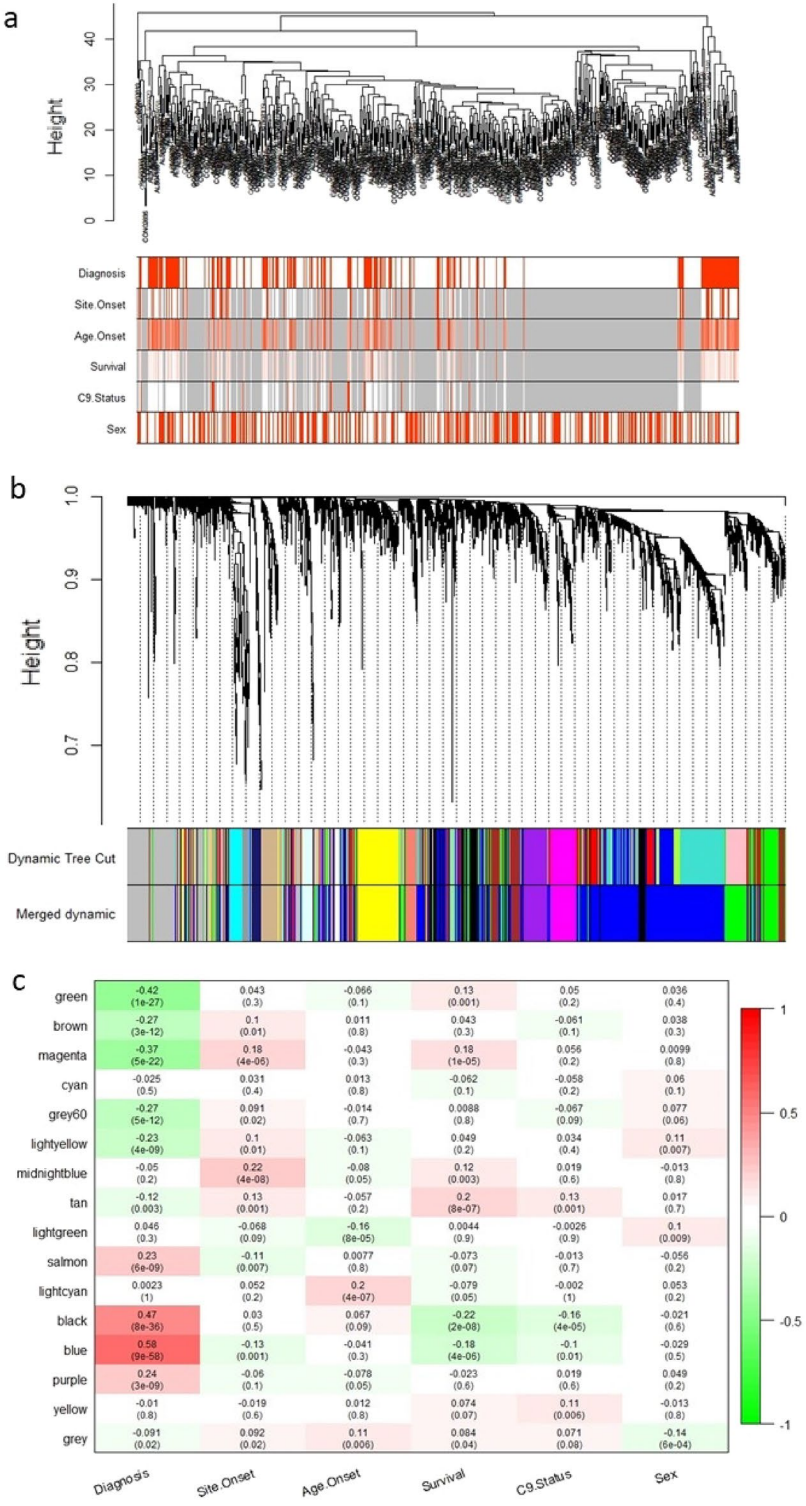
(**a**) Sample dendrogram with traits heatmap. Red is ALS in diagnosis, female in Sex, Spinal in site.onset, Yes in C9 state, the higher value in age.onset and survival. Gray is equal to NA. (**b**) Clustering dendrogram of genes, dissimilarity is based on topological overlap, together with assigned module colors. As a result, 20 co-expression modules were constructed and was shown in different color. Merging the modules with high correlated eigengenes, resulted in 16 modules. The number of genes in each module were listed in Table S2. (**c**) Module-trait associations (ALS vs Control). Each row corresponds to a module eigengene, column to a trait. Each cell contains the corresponding correlation and P-value. The table is color-coded by correlation according to the color legend.

First, the sample clustering was obtained using hierarchial clustering. The sample outliers were detected and removed by pruning. The sample dendrogram was plotted to all traits (Supplementary Fig. 3, Fig. 1a). When the power value was set to 8, the independence degree was up to 0.8 (Supplementary Fig. 4). By raising the matrix of expression to soft-thresholding (β) of 8, TOMs and consequently dissTOMs were obtained. The cluster dendrograms were created based on the TOMs with a minimum cluster size of 30 and modules with a high correlated eigengenes have been merged with a threshold value of 0.2 (Supplementary Fig. 5).

In order to identify modules that were significantly related to the evaluated clinical features, the expression profiles of each module were summarized as the eigenvector correlated to the first main component of the expression matrix using the Module Eigengene (ME). Values of gene significance (GS) were used to calculate the association of individual genes with the ALS. In addition, the Module Membership (MM) was defined as the ME correlation and the gene expression profile for each module. When the GS and MM were strongly associated, the most important (central) elements in the modules were closely related to the trait and can also be considered as hub-genes.

In total, 16 distinct co-expression modules were identified; each module was assigned a unique number and a unique color (Fig. 1b). The irrelevant genes were allocated to module0 or gray module. The correlation analysis was performed between modules and clinical traits. As shown in Fig. 1c and Supplementary Table S2, the blue, black and green modules with 1361, 257, and 501 genes, respectively, had the most correlation with clinical state of ALS (R = 0.58, Pval = 9e−58 and R = 0.47, Pval = 8e−36, R = −0.42, Pval = 1e−27; respectively). Other modules including magenta, brown, gre60, light yellow, salmon, and purple were correlated to ALS diagnosis, but the R values indicated a poor correlation (R < 0.4). Because of the positive relation with ALS, we selected the blue and black ALS-related modules for further analysis. Meanwhile, the both modules were correlated to lower survival of patients (R = −0.18, Pval = 4e−06 and R = −0.22, Pval = 2e−08, respectively) and to some extent, negative genetic testing for C9orf72 (R = −0.1, Pval = 0.01 and R = −0.16, Pval = 4e−5, respectively). Magenta module also, were correlated to spinal site of onset (R = 0.18, Pval = 4e−06; respectively). There was no significant correlation between the modules and the other clinical features.

### Preservation test of modules

**Figure 2:**
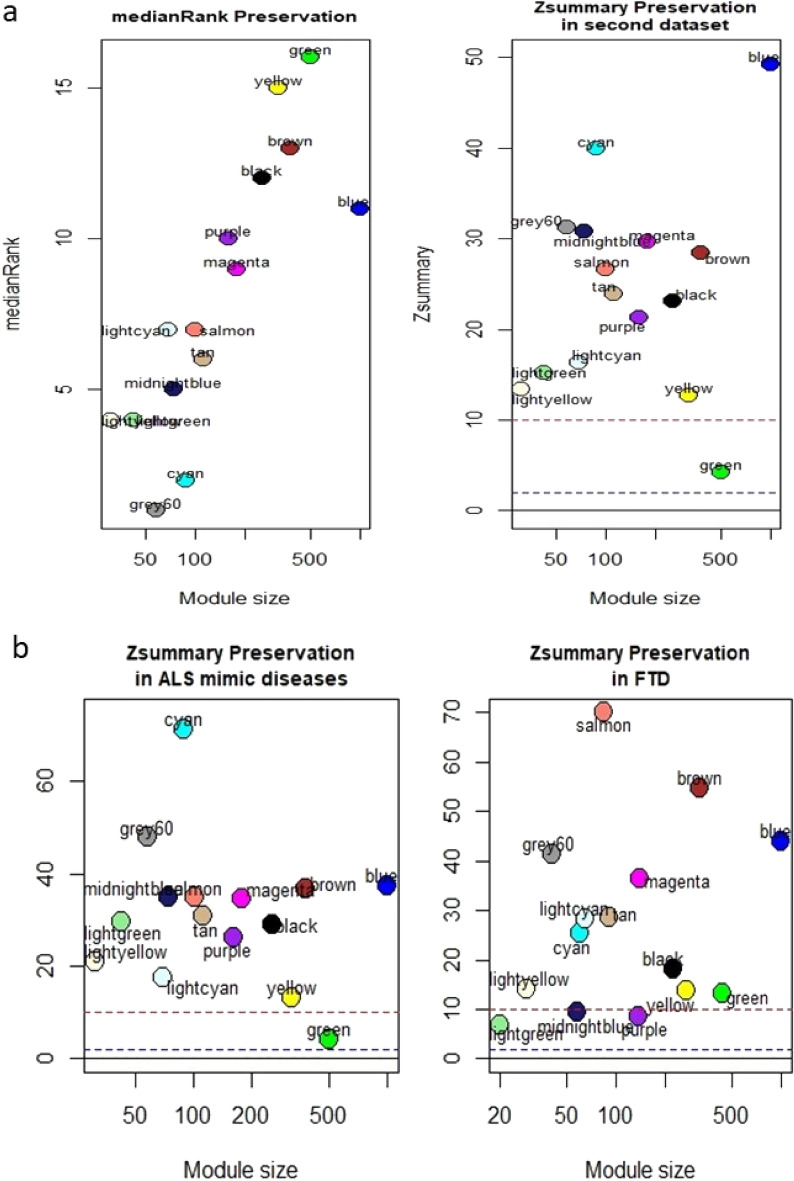
The median rank and Z-summary statistics of the module preservation in validation dataset and (**a**) and the Z-summary of module preservation in ALS mimic diseases and FTD (**b**). The dashed blue and red lines in the graphs indicate the thresholds Z = 2 and Z = 10, respectively. These horizontal lines indicate the Zsummary thresholds for strong evidence of conservation (> 10) and for low to moderate evidence of conservation (> 2). All the modules are strongly preserved.

In order to evaluate the preservation of ALS-related modules in the validation dataset (ALS and control subjects of dataset GSE112680), we assessed the module preservation using z-summary. According to the results of module preservation analysis, all modules except green were strongly preserved (preservation Z‑score > 10) between two datasets (Fig. 2a). Subsequently, we tested whether the ALS-related modules preserved in ALS-mimic and FTD diseases using the module preservation method in datasets GSE112680 (ALS-mimic and control subjects) and GSE140830, respectively. The results indicated that green module is non-preserved in mimic diseases, whereas lightgreen, midnightblue, and purple modules are poorly preserved in FTD patients (Fig. 2b). Since green modules were non-preserved in validation dataset too, it could not be considered as ALS-mimic related module. While, Light green, midnightblue, and purple modules were considered as FTD-related modules.

### Enrichment analysis of the main modules

**Figure 3:**
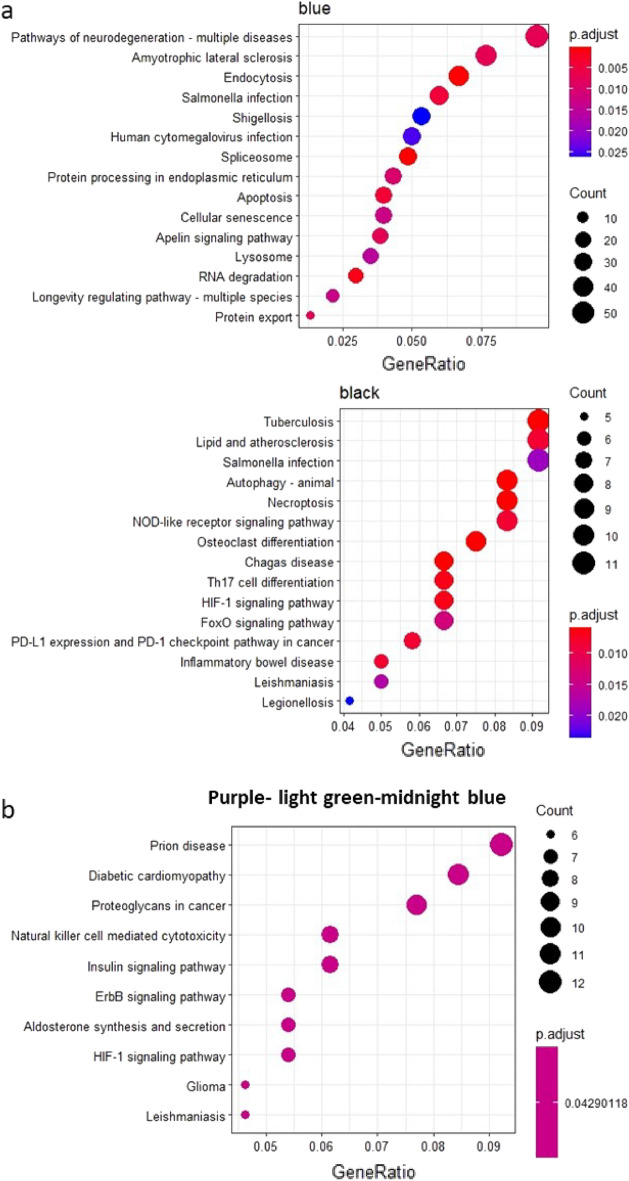
KEGG enrichment analysis of main modules. (**a**) The top 15 terms of KEGG enrichment in ALS-related modules; blue and black, separately. (**b**) Enrichment analysis of FTD-related modules including purple, light green, and midnight blue.

Enrichment analysis was performed for the genes in the significant co-expression modules. Genes in the blue module were the most enriched in KEGG pathways: ‘pathways of neurodegeneration-multiple diseases’, ‘amyotrophic lateral sclerosis’, and ‘endocytosis’. However, the pathways related to ALS such as ‘apoptosis’, ‘cellular senescence’, ‘lysosome’ are of significant enriched terms in the blue module. The enrichment of inter-connected genes in black module resulted in terms related with ALS such as: ‘autophagy-animal’, ‘necroptosis’, and ‘foxO signaling pathway’ (Fig. 3a). Among the other ALS-related modules, salmon and brown modules were enriched in ‘amyotrophic lateral sclerosis’, too (Supplementary Table S3).

The genes in modules non-preserved in FTD disease, including lightgreen, midnightblue, and purple modules, were enriched in ‘prion disease’, and pathways related to metabolic disorders such as ‘diabetic cardiomyopathy’, ‘insulin signaling pathway’, and ‘aldosterone synthesis and secresion’ (Fig. 3b).

### Identification of main modules and hub genes

As mentioned above, blue and black were defined as ALS-related modules, while purple; lightgreen and midnightblue were considered as FTD-related modules. When the genes in each module met the criteria of MM > 0.8, they were considered as the module's hub genes. The lists of hub genes according to modules have provided in Supplementary Table S4.

Subsequently, the DEGs obtained from previously studies were overlapped with the hub genes of the ALS-related modules and presented using a Venn diagram (Supplementary Fig. 6). Thirty overlapping genes were identified as follows: CNBP, AQP9, TAF7, TBK1, CRLS1, CDKN1B, ANAPC13, SH3GLB1, ASNSD1, MNDA, YPEL5, CLDND1, CHUK, HBP1, MKLN1, C1orf52, QPCT, SLC35A1, RGS18, GPBP1, STX3, VEZF1, TMEM71, CRBN, HIST1H2AC, PPP2R3C, TMEM126B, SAMD9, VNN2, and STXBP3.

### Protein–protein interaction network

**Figure 4:**
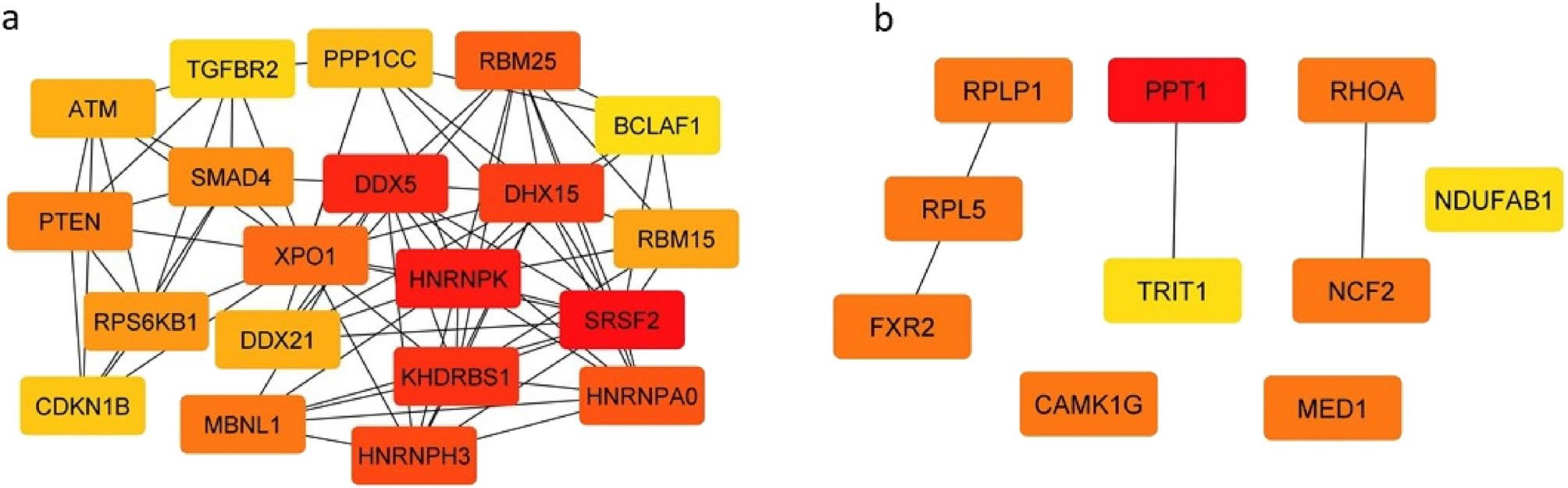
Protein–protein interaction (PPI) network analyzed by cytoHubba app of Sytoscape. (**a**) Top 20 key genes of the hub genes in ALS-related modules. (**b**) Top 10 key genes of the hub genes in FTD-related modules. The key genes explored according to MMC algorithm of cytoHubba. Darker red of nodes presents higher MCC score.

We used STRING to establish a PPI network with 264 nodes and 571 edges from 265 hub genes in blue and black modules. The PPI data were imported into Cytoscape software (version 3.9.1) and CytoHubba app was employed to predict important nodes or subnetworks based on the MCC (Maximal Clique Centrality) algorithm. The top 20 genes (SRSF2, HNRNPK, DDX5, KHDRBS1, DHX15, HNRNPH3, HNRNPA0, RBM25, XPO1, MBNL1, PTEN, SMAD4, RPS6KB1, RBM15, ATM, DDX21, PPP1CC, CDKN1B, TGFBR2, BCLAF1) are shown in Fig. 2B. Further, a PPI network was made for FTD-related modules. Ten top genes obtained from cytoHubba algorithm are presented in Fig. 4.

### Validation of hub genes using the LASSO logistic analysis

**Figure 5:**
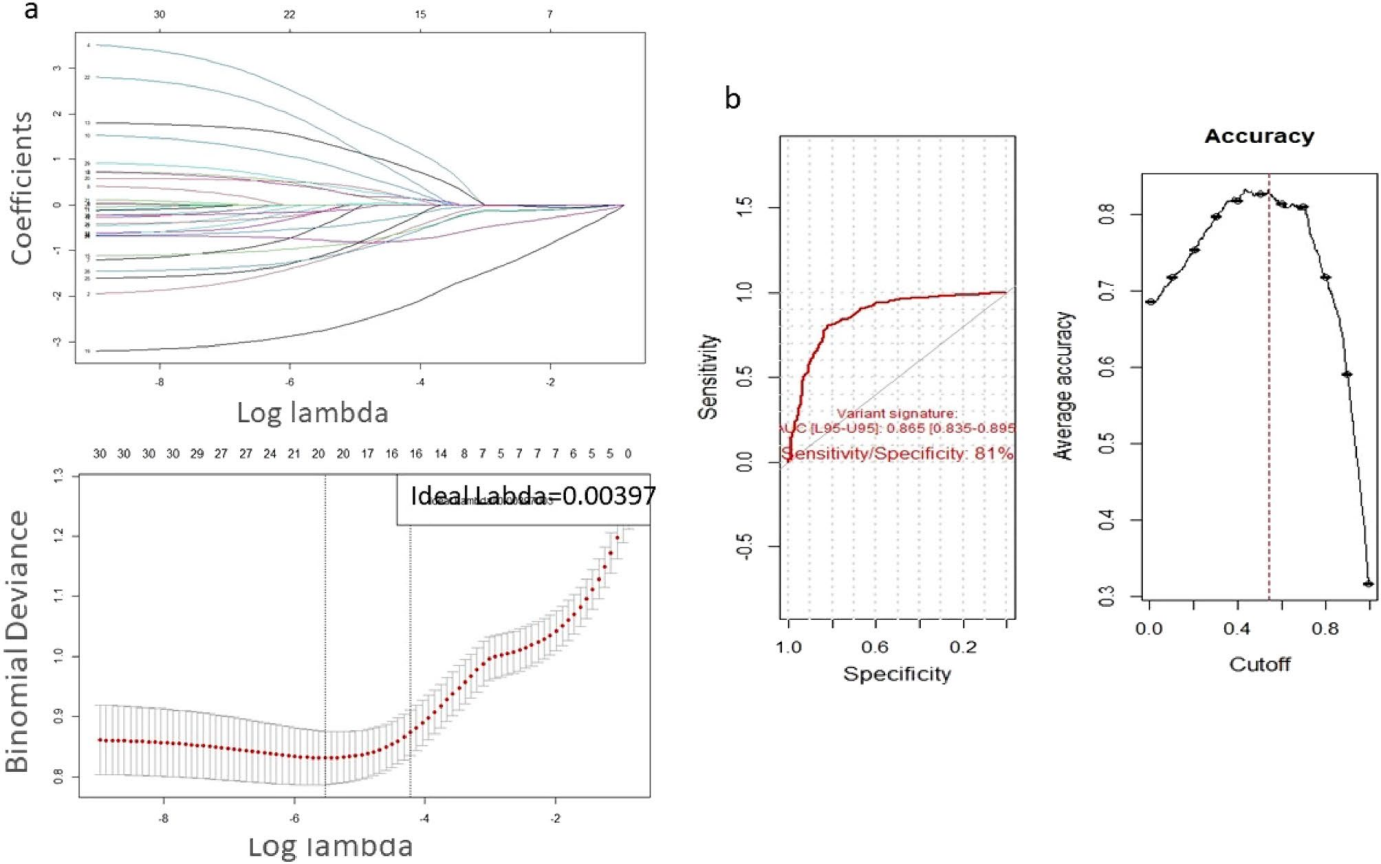
(**a**) The Lasso.model and cv.Lasso model including the selection of the optimal parameter lambda (λ). The optimal λ-value was 0.0021. (**b**) Five genes were finally selected. The ROC and optimum accuracy of 5-genes set for diagnosis of ALS. The values of AUC imply the 5-genes set is an excellent predictor for ALS disease.

We screened out the significant genes of the ALS-related modules according to Eigengene-based module connectivity or module membership (MM > 0.8) and gene significance as hub genes related to ALS diagnosis GC > 0.5). Then, the top 30 hub genes were subjected to LASSO regression analysis. Based on the tenfold cross-validation, the remaining genes with nonzero LASSO coefficients were obtained (Fig. 5). Moreover, the final LASSO was calculated based on the optimal lambda value.

The 5 top genes with nonzero coefficients including BCLAF1 (95%CI 6.61–78.6; Pval = 8.57e−07), GNA13 (95%CI 1.76–9.98; Pval = 0.001), ARL6IP5 (95%CI 1.37–6.04; Pval = 0.005), ARGLU1 (95%CI 0.08–0.32; Pval = 1.53e−07), and YPEL5 (95%CI 0.08–0.38; Pval = 1.93e−07) were considered as key diagnostic genes. The AUC of the 5-genes set indicated that they have an excellent value for discriminating ALS from control subjects.

## Discussion

Despite the progression made in ALS genetics, little is known regarding the complex pathogenic mechanisms leading to motor neuron degeneration and the clinical entity of this debilitating disease. The gene expression-based studies have improved our understanding about the genetic perturbation associated with ALS, nevertheless the findings are strongly affected by the disease heterogeneity and sample-specific variation, making the result inconsistent across independent studies. One solution to address the problem of heterogeneity involves meta-analysis of multiple independent studies that increase the sample size and statistical power^27^. Another approach involves the study of the interaction among groups of genes, i.e. modules, which are investigated at the systems-level to identify genes associated with common function in a given biological state^28^. By network analysis, i.e. WGCNA, genes with highly correlated expression are grouped into modules yet correlate with distinct biological pathways. Here, network analysis identified the blue module that were strongly correlated with ALS trait (r = 0.58, Pval = 9e−58). The genes related to ALS including C9orf72, TARDBP, CHMP2B, TBK1, VCP, DCTN1, OPTN, FIG4, ATXN2, BCL11B and PRPH were in the blue module. The blue module was enriched in pathways related to ALS, including: ‘Amyotrophic lateral sclerosis’, ‘Pathways of neurodegeneration-multiple diseases’, ‘Endocytosis’, ‘Lysosome’, ‘Apoptosis’, ‘FoxO signaling pathway’ and ‘Autophagy-animal’. ‘Amyotrophic lateral sclerosis’ was also enriched in brown and salmon modules. Pathways such as ‘Autophagy’ and ‘Lysosome’ were the common terms in multiple modules.

We also demonstrated a new 5-genes signature for diagnosis of ALS that was assigned through logistic LASSO regression analysis. The LASSO regression is a current widely used model for reducing the number of features, estimating of regression parameters, and selecting features especially in huge datasets. Furthermore, the estimation of area under ROC curve (AUC) demonstrated the 5-genes set as an excellent predictive gene set for ALS diagnosis. All the genes including BCLAF1, GNA13, ARL6IP5, ARGLU1, and YPEL5 are involved in apoptosis. This is in accordance with the gene ontology term of ‘programmed cell death’ that was significantly enriched for genes differentially expressed between ALS and control subjects in the study of Van Rheenen^15^.

Bcl-2 associated factor1 (BCLAF1) is a binding protein that was originally identified to interact with anti-apoptotic Bcl-2 family member E1B19K, and induce apoptosis and suppress transcription^29^. Initial research demonstrated that BCLAF1 also plays a role in multiple biological processes that are not related to actions of Bcl2 family members, including RNA processing, skeletal muscle differentiation, DNA damage response, and autophagy. Notably, there is evidence that BCLAF1 could serve as a strong autophagy inducer by displacing beclin-1 from BCL2 in myeloma cells^30^. Another study reported that BCLAF1-mediated autophagy could prevent cell apoptosis and increase cell proliferation in HCC cells^31^. The role of BCLAF1 in ALS is not yet evaluated. However in dopamin neurons of patients with Parkinson, it is indicated that BCLAF1 is downregulated which is associated with programmed cell death and mitochondrial function^32^. A recent study showed that cardiac ischemia–reperfusion (I/R) injury can elicit BCLAF1 overexpression followed by upregulation of apoptosis-related proteins suggesting BCLAF1 been a detrimental factor in cardiac I/R injury^33^.

Guanine nucleotide-binding protein subunit alpha-13 (GNA13 /Gα13) is a member of the G protein family. In general, Guanine nucleotide-binding proteins (G proteins) are involved in transmembrane signal transducing and alpha subunits play a critical role in signal transducing by binding to GTP and transforming it to GDP^34^.

GNA13 have been particularly associated with tumor invasion and metastasis in multiple types of cancer through RhoA/ROCK^35,36^ and PI3/AKT signaling pathways^37^. GNA13 is overexpressed in tumors and closely associated with an aggressive phenotype and poor patient's prognosis^38^. GNA13, also negatively regulates BCL-2 and inhibits PI3K-AKT signaling pathway, whereas its deficits impairs apoptosis in germinal center B-cells^39,40^. In neuronal cells, GNA13 is involved in neurite outgrowth and synaptic function through Rho signaling pathway. It is indicated that GNA13 is downregulated in prefrontal cortex of schizophrenia brain and may have negative effect on synaptic plasticity by suppression of GNA13-ERK pathway^41^. A recent study showed that GNA13 is upregulated in Alzheimer's disease and increases as the disease worsens^42^. The dysregulation of GNA13 in ALS is reported previously^43,44^, however the affected mechanisms is unknown.

ADP ribosylation factor-like GTPase 6 interacting protein 5 (ARL6IP5), also known as JWA gene encodes a microtubule-associated protein that contains a prenylated Rab acceptor motif. It regulates intracellular protein transport and oxidative stress^45^. ARL6IP5, also has been shown to induce endoplasmic reticulum (ER) stress-mediated apoptosis in osteoblast cells^46^, involve in TRAIL-triggered cell apoptosis in gastric cancer^47^, and play a protective role against DNA damage induced by oxidative stress^48^. The findings suggest that ARL6IP5/JWA play a critical role in oxidative stress-induced DNA damage. The role of ARL6IP5 in the pathogenesis of ALS has never been investigated. However, there is an increasing data that indicated ARL6IP5 has a neuroprotective role in DA neuronal cells via modulating intracellular redox and NF-κB signaling pathway^45^, regulating Nrf2^49^, and modulating GLT-1 expression and glutamate uptake in PD models^50^.

Arginine and glutamate rich 1 (ARGLU1) is a highly evolutionarily conserved with a dual function. The glutamate rich C-terminal is able to co-activate glucocorticoid receptor (GR) and the arginine rich N-terminus modulate RNA splicing RNAs^51^. Glucocorticoids are expressed in multiple regions of the brain. During chronic stress, glucocorticoids bind to GR and activate them. There is evidence that chronic stress is associated with neurodegenerative diseases. Clinical studies suggest that exposure to chronic stress followed by elevated GC levels can be a causal factor for neurodegenerative induction and progression^52^. Magomedova et al. in a study have identified ARGLU1 as a GR coactivator and RNA binding protein that is involved in the both layers of gene regulation including transcription and alternative splicing in neuronal cells^51^. Although, it has not been revealed the role of ARGLU1 in ALS, it has indicated in some pathological conditions that ARGLU1 serves as an anti-apoptotic factor. For example, targeting of ARGLU1 by miRNAs cause induction of apoptosis and inhibition of proliferation in hypertrophic scar^53^, uterine leiomyoma^54^ and breast cancer^55^.

YPEL5 is a member of the YPEL gene family that is highly conserved in the eukaryotic species and apparently involved in a certain cell division-related function. YPEL5 protein has been shown to be involved in the cell cycle progression^56^. Human YPEL5 plays pro-apoptotic role and is also involved in DNA damage-induced apoptosis^57^. Nonetheless, the dysregulation of the YPEL5 in ALS patients or models were not reported. YPEL5 were ubiquitously expressed in human tissues. YPEL5 is localized in the nucleus and centrosome, suggesting the conservation of a pro-apoptotic role of YPEL5 in DNA damage-induced apoptosis^57^.

ALS and FTD with common genetic and clinical traits giving rise to an ALS/FTD spectrum that indicate some common pathological mechanisms leading to either of these neurological disorders. However, the pattern of two-disease onset is quite different. To identification of genes with different co-expression patterns in two diseases leading to distinct the different types of neurons involved, we analyzed the non-preserved modules between two expression datasets. Three modules with genes were non-preserved and considered as FTD-related modules. Enrichment analysis of these modules revealed the genes are involved in some metabolic impairments and prion disease. It has previously been shown that metabolic dysfunction, including the insulin-signaling pathway, is associated with cognitive impairment, including dementia. For example, there is a link between insulin resistance and cognitive impairment^58^. In this regard, the hub genes of FTD-related modules including HEPB2^59^, CAMK1G^60^, ADRA2G^61^, C22orf132^62^ are associated with cognitive impairment. Increasing evidence points to closely relevant between insulin signaling impairment and pathologic mechanisms of cognitive dysfunction.

Insulin function in neurons is very important and can affect many biological functions including blood glucose and metabolism regulation^58^. The association of ALS and insulin resistance was evidenced in past researches. Several studies have evaluated the association between insulin resistance and ALS and reported conflicting results from a protective effect up to a risk factor for developing ALS. A recent review study has concluded that metabolism changes occurred in FTD and ALS can be the result of neurodegeneration trend with a secondary effect of destroying the vital brain regions, especially in FTD patients. Giving the ALS-FTD continuum, the ALS patients without cognitive deficits have a different pattern of energy metabolism rather than FTD, whereas ALS patients with cognitive deficits have an increased BMI and eating changes (hyperphagia) compared with patients with ALS without cognitive deficits. The hyperphagia go to be sever in FTD patients^21^.

Meanwhile, preservation analysis of ALS-mimic patients' gene expression did not result in any non-preserved module that could be considered as MIM-related module. The result was consistent with the original study by van Rheenen et al. who did not find differentially expressed genes between ALS and ALS-mimic patients. This may be due to the large variety of patients who had participated in the study as ALS-mimic. There were 75 ALS-mimic patients with 25 different forms of diagnosis. Significant results were likely to be obtained if more participants had been selected with less variation in ALS-mimic disorders.

## Conclusion

In conclusion, the current study combined WGCNA algorithm and LASSO logistic regression to screen potentially diagnostic biomarkers associated with ALS. We identified ARGLU1, CHMP2B, GNA13, SMAD4, VPS26A, and YPEL5 genes related to ALS. Moreover, we found autophagy may be involved in pathogenesis of ALS. The genes can be detected in blood and should be further studied as possible biomarkers of disease and drug development.

## Supplementary Information

Supplementary Information 1.

Supplementary Information 2.

## Data Availability

The datasets analyzed during the current study are available in the European Nucleotides Archive with accession number GSE112676, GSE112680, and GSE140830.
