# Author Correction: Identification of potentially functional modules and diagnostic genes related to amyotrophic lateral sclerosis based on the WGCNA and LASSO algorithms

**DOI:** 10.1038/s41598-022-27367-5

**Published:** 2023-01-03

**Authors:** Narges Daneshafrooz, Masumeh Bagherzadeh Cham, Mohammad Majidi, Bahman Panahi

**Affiliations:** 1 Department of Neuroscience, Faculty of Advanced Technologies in Medicine, Iran University of Medical Science, Tehran, Iran; 2 School of Rehabilitation Science, Iran University of Medical Science, Tehran, Iran; 3 Neuromusculoskeletal Research Center, Physical Medicine and Rehabilitation Department, Firoozgar Hospital, School of Medicine, Iran University of Medical Science, Tehran, Iran; 4 Department of Biotechnology, Faculty of Agriculture, Azarbaijan Shahid Madani University, Tabriz, Iran; 5 Department of Genomics, Branch for Northwest & West Region, Agricultural Biotechnology Research Institute of Iran (ABRII), Agricultural Research, Education and Extension Organization (AREEO), Tabriz, Iran

Correction to: *Scientific Reports* 10.1038/s41598-022-24306-2, published online 22 November 2022

The original version of this Article omitted an affiliation for Narges Daneshafrooz. The correct affiliations are listed below.

Department of Neuroscience, Faculty of Advanced Technologies in Medicine, Iran University of Medical Science, Tehran, Iran.

School of Rehabilitation Science, Iran University of Medical Science, Tehran, Iran.

The original Article has been corrected.

