# Supplementary material for: Identification of potentially functional modules and diagnostic genes related to amyotrophic lateral sclerosis based on the WGCNA and LASSO algorithms
Source: Sci Rep. 2022 Nov 22;12:20144. doi: 10.1038/s41598-022-24306-2 (PMC9684499; doi:10.1038/s41598-022-24306-2)
Supplement: Supplementary file 1 — Supplementary Information 1. [file 41598_2022_24306_MOESM1_ESM.docx]

**Supplementary File**

**Identification of potentially functional modules and diagnostic genes related to Amyotrophic Lateral Sclerosis based on the WGCNA and LASSO Algorithms**

**Supplementary Table S1.** Briefly sample information.

|  | **GSE112676** | | **GSE112680** | | | **GSE140830** | |
| --- | --- | --- | --- | --- | --- | --- | --- |
| **Platform** | GPL6947 | | GPL10558 | | | GPL15988 | |
| **Diagnosis** | ALS | CON | ALS | CON | MIM | FTD | CON |
| **Number** | 233 | 508 | 164 | 137 | 75 | 172 | 281 |
| **Sex (f/m)** | 90/143 | 230/278 | 68/96 | 58/79 | 17/58 | 85/87 | 156/125 |
| **Age (year)** | - | - | - | - | - | 64.4 | 71.5 |
| **Site of onset (s/b)** | 143/90 | - | 113/51 | - | - | - | - |
| **Age at onset (year)** | 62.37  (24.16-88.49) | - | 61.90  (23.12-86.44) | - | - | - | - |
| **Survival (year)** | 3.01  (0.34-16.5) | - | 2.69  (0.40-13.58) | - | - | - | - |
| **C9orf72 state (Y/N)** | 24/209 | - | 40/124 | - | - | - | - |

ALS: Amyotrophic lateral sclerosis, CON: Control subjects, MIM: ALS-mimic diseases, FTD: Frontotemporal dementia.

C9orf72 hexanucleotide expansion has tested in ALS patients.

**Supplementary Table S2.** The size of modules.

| **Primary modules** | | **Merged modules** | |
| --- | --- | --- | --- |
| **Modules** | **Module size** | **Modules** | **Module size** |
| Green | 292 | Green | 501 |
| Pink | 209 |  |  |
| Brown | 380 | Brown | 380 |
| Magenta | 178 | Magenta | 178 |
| Black | 257 | Black | 257 |
| Purple | 162 | Purple | 162 |
| Red | 280 | Blue | 1361 |
| Turquoise | 550 |  |  |
| Blue | 400 |  |  |
| Greenyellow | 131 |  |  |
| Lightgreen | 42 | Lightgreen | 42 |
| Salmon | 101 | Salmon | 101 |
| Lightcyan | 69 | Lightcyan | 69 |
| Cyan | 88 | Cyan | 88 |
| Grey60 | 59 | Grey60 | 59 |
| Yellow | 322 | Yellow | 322 |
| Lightyellow | 32 | Lightyellow | 32 |
| Midnightblue | 74 | Midnightblue | 74 |
| Tan | 113 | Tan | 113 |
| Grey | 458 | Grey | 458 |

**Supplementary Table S4.** The modules related to diagnosis traits and their hub genes.

| **Trait** | **Main module(s)** | **Hub genes** |
| --- | --- | --- |
| ALS-related | Blue | ARGLU1, BCLAF1, PPP2CA, CNIH, SMAD4, YPEL5, PCNP, PAPD4, TOR1AIP1, ZMPSTE24, ARL6IP5, SNRK, GNA13, HIGD1A, GPBP1, XPO1, C6orf62, VPS26A, DCP2, EIF4G2, ZFAND5, PJA2, PCMT1, PQLC3, TMED5, SMG1, MAP2K1IP1, CENTB2, ROD1, ACBD3, PPP1CC, DHX15, SLC35A1, ELF1, KIAA1370, CLK1, EFHA1, CPNE3, PTPN12, ZCCHC7, CD47, GHITM, PAPOLA, TMEM66, CRBN, FBXO33, TAF7, VEZF1, REEP5, OBFC2A, LANCL1, RBM25, RPS6KB1, UBE2N, RSBN1, RCBTB2, LEPROTL1, ADSS, TBK1, VPS4B, FOXN2, PRNP, RAB8B, HMGN4, CPD, IVNS1ABP, PCMTD2, VPS29, DNAJA2, CDKN1B, C1orf19, BTAF1, JMJD1C, PRKAR1A, RBM15, KIAA1600, ANGEL2, BIRC2, PELI1, PPCS, SAMD9, CD46, PCMTD1, TOMM20, C7orf23, USP38, CHMP2B, GOLPH3, PTPLB, DERL1, NCK1, C14orf106, BNIP2, CRLF3, TMEM123, CNBP, C14orf100, ACTR2, LOC730432, SLMAP, MON2, CAB39, ATP6V1G1, CRLS1, TLE4, ATM, GNG2, C1orf52, DEK, OCIAD1, RAB21, VPS36, ZNF217, RB1CC1, RNGTT, TMEM188, ABHD3, ASNSD1, ZDHHC17, ABCE1, ANKRD12, STXBP3, TMED7, SFRS2, FEZ2, NPTN, COQ10B, C14orf138, GCA, NSMAF, CCDC14, RAB11A, KIDINS220, ARL8B, EBI2, RCOR3, NCOA7, SCYL2, UBE2E1, DEGS1, C20orf30, SMEK2, CALM2, ENOPH1, SUZ12, MAP3K8, CD44, BRP44L, PRDX3, FAM96A, OXR1, MBNL1, TMEM126B, ACADM, ZNF800, C2orf30, FAM120A, EFR3A, TGFBR2, GNAI3, C19orf2, MAT2B, LEMD3, PURB, DDX21, SFT2D1, GMFB, KIAA0528, RNF145, CD58, CCPG1, WDFY1, AMD1, ARID5B, ARL5A, TFAM, WASPIP, KLHL9, FEM1C, ALG6, THOC7, HNRPH3, C1orf55, NDUFB5, TMEM209, BTG1, EPS15, SERINC1, GGNBP2, PRKAA1, CLINT1, UBL3, CLDND1, RGS18, MTSS1, TRAM1, NUP160, KLF9, SLC38A2, TIPARP, RSBN1L, KHDRBS1, PTGER4, HNRNPA0, RBM17, LMBRD1, DDX5, ZEB2, CNOT8, RAB5A, CHUK, ANKRD10, ANAPC13, BAZ2B, RAP2A, SLC30A9, MKLN1, C6orf111, CHD1, SLC35A5, COPB1, C1orf131, C14orf32, DDX46, PPP2R3C, C1orf59, ZNF25, USP15, PTGER2, TBC1D15, C1orf63, ARL2BP, PELI2, HNRPK, RAB22A, PDCD6, RBM22, NIP7, SOLH, BAP1 |
|  | Black | QPCT,SH3GLB1,FPR2,DUSP1,MXD1,NDEL1,AQP9,CHMP1B,PTEN,EVI2B,IFNGR2,NAMPT,SDCBP,STX3,STX11,NUMB,IFNGR1,FOS,HIST1H2AC,RAF1,HBP1,MNDA,TMEM71,VNN2,ATP6V1A |
| FTD-related | purple | HEBP2, ACSL1, ATF4, PHF23, FTH1, SAP30L, TMEM154, PHIP, RNF20, F11R, NDUFAB1, YWHAG, NMI, PRCP, TLR1, PPARBP, PYGL, IMMT, HDAC4, GIMAP1, PPT1, TRIP12, C14orf166, OSBPL2, C19orf50, C19orf43, CITED2, PKN2, CORO1C, C6orf115, SAT2, INTS8, RHOA, RNF149, NCF2, TXNIP, NT5C2, B4GALT1, TRIT1, RPLP1, RTN3 |
|  | midnightblue | ADRA2C, CAMK1G, CCDC52, HOXC10, C20orf132, C22orf30, LOC652968, ARTN, LOC152578, TYW1, TRPA1, RNF213, RTF1, PKP2, FXR2, MUC6, G6PC, KIR3DL2, DPYSL5, OR51S1, LOC648526, NRSN2 |
|  | lightgreen | RPL5, LOC388654, LOC643284, LOC440927, RPL37A, LOC285053 |


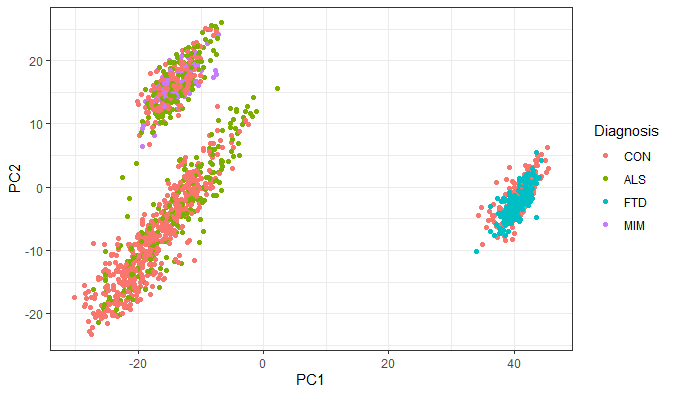


**Supplementary Figure 1.** Principle component analysis of dataset GSE112681 (including GSE112676 and GSE112680) and dataset GSE140830.

**
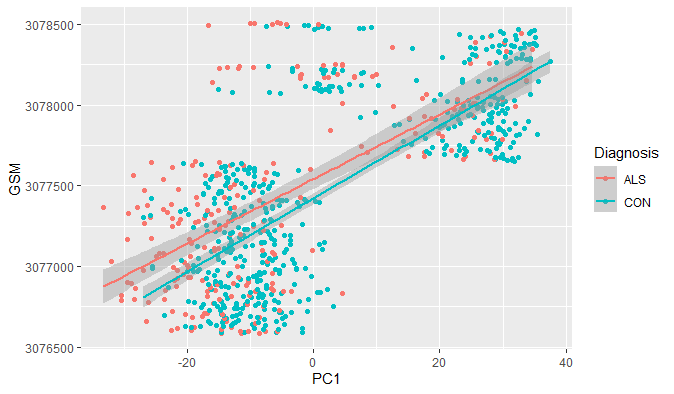

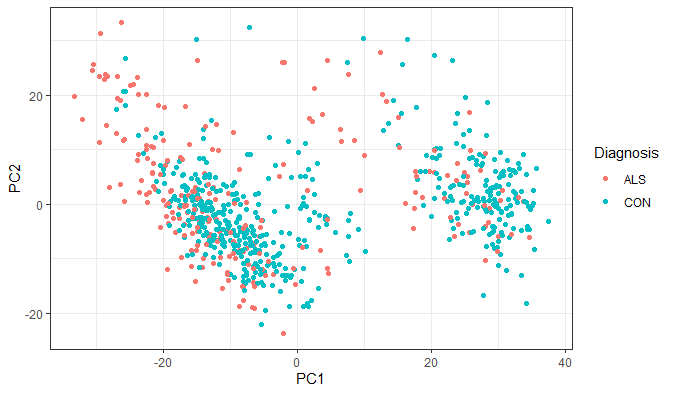
a b**

**c d**


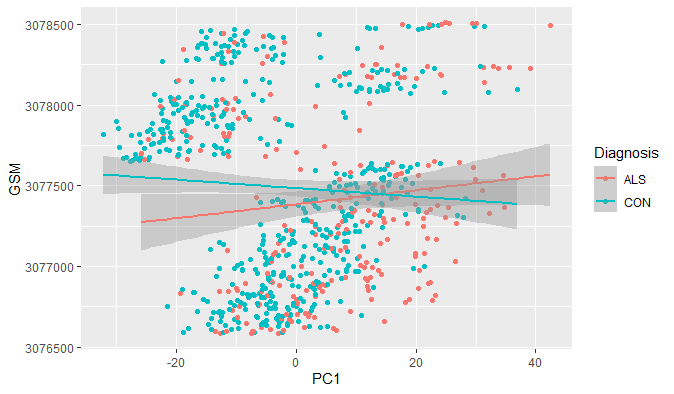

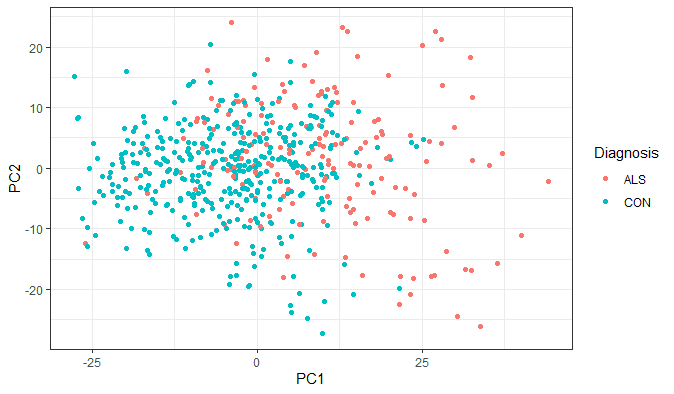
**Supplementary Figure 2.** Principle component analysis (PCA) of dataset GSE112676. a) an obvious batch effect is seen in the dataset. b) there is a significant correlation between GSM indices and the PC1. c) PCA of adjusted data shows the correction of the intra-platform batch effect. d) The correlation between GSM indices and PC1 has removed too.


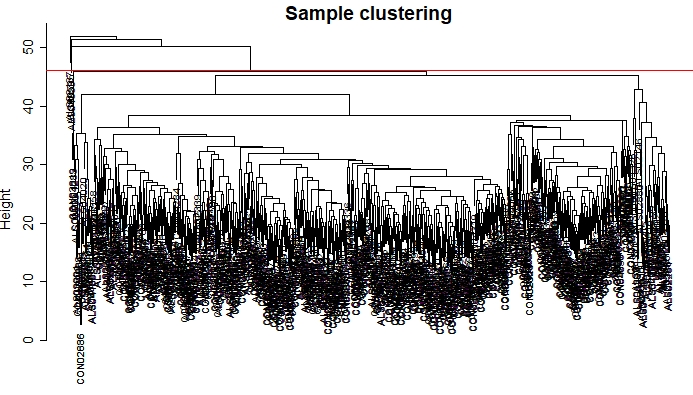


**Supplementary Figure 3.** Sample clustering and pruning to remove outliers in dataset GSE112676.


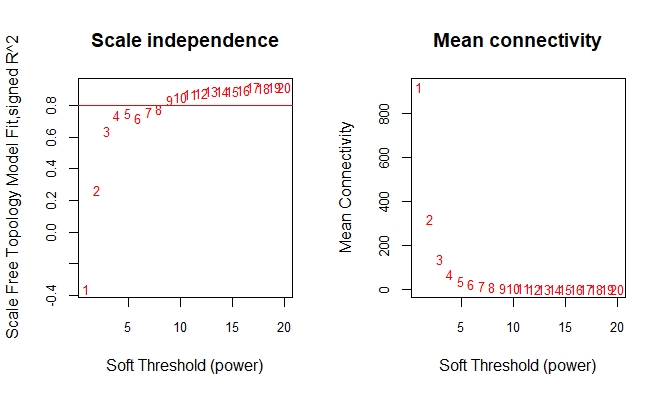


**Supplementary Figure 4.** Analysis of network topology for various soft-thresholding powers. The left panel shows the scale-free fit index (y-axis) as a function of the soft-thresholding power (x-axis). The right panel displays the mean connectivity (y-axis) as a function of the soft-thresholding power (x-axis). By setting the power to 8, the independence degree was up to 0.8.

**a**


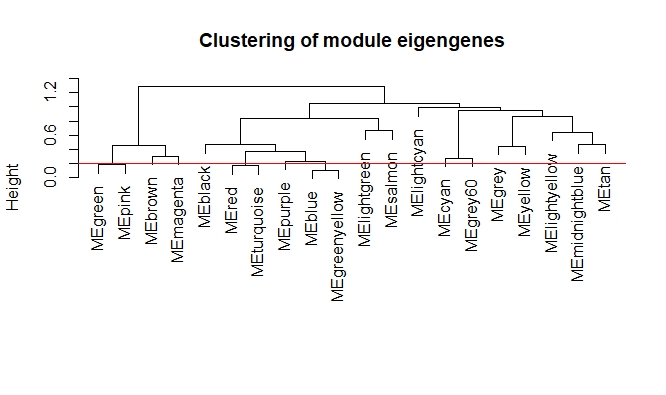


**b**


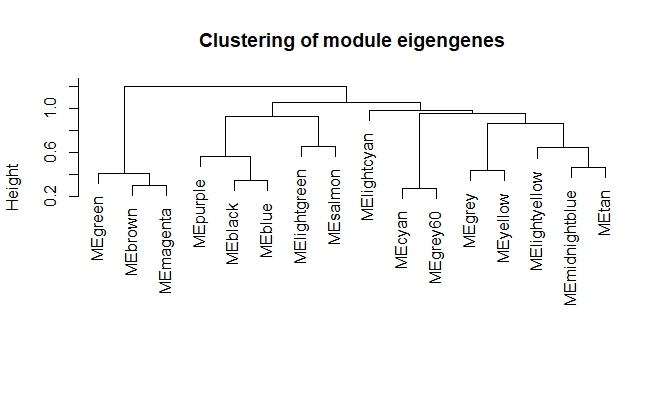


**Supplementary Figure 5.** Clustering of module eigengenes, a) before and b) after merging modules with a high correlated eigengenes.


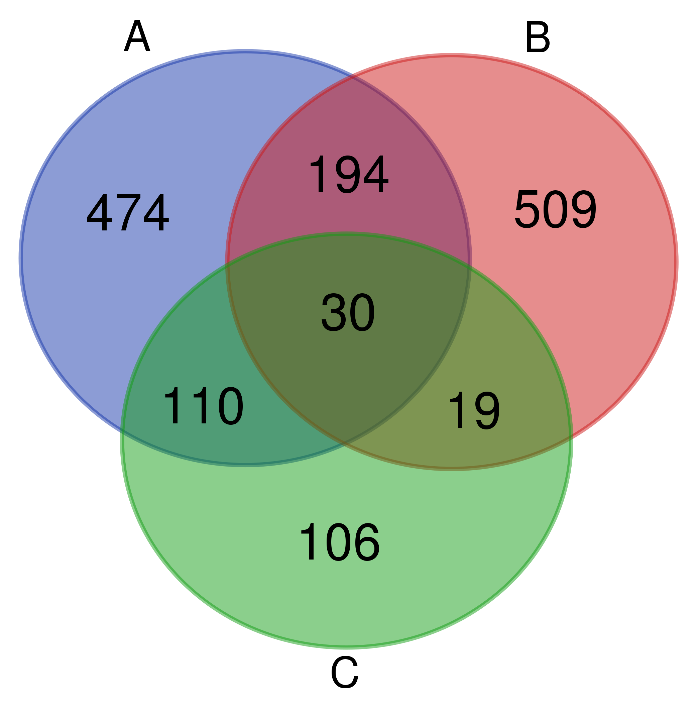


**Supplementary Figure 6.** Venn diagram shows the identified genes overlapping between differentially expressed genes of Van Rheenen study (A), and Swindell study (B) and the hub genes of ALS-related module in the present study. There were 30 intersected genes between three studies.
